# Cancer prevalence in the United Kingdom: estimates for 2008

**DOI:** 10.1038/sj.bjc.6605148

**Published:** 2009-06-30

**Authors:** J Maddams, D Brewster, A Gavin, J Steward, J Elliott, M Utley, H Møller

**Affiliations:** 1 Kings College London, Thames Cancer Registry, 1st Floor Capital House, 42 Weston St, London SE1 3QD, UK; 2 Scottish Cancer Registry, Area 155, Gyle Square, 1 South Gyle Crescent, Edinburgh EH12 9EB, UK; 3 Northern Ireland Cancer Registry, Queen's University Belfast, School of Medicine Dentistry and Biomedical Sciences, Mulhouse Building, Grosvenor Road, Belfast BT12 6BJ, UK; 4 Welsh Cancer Intelligence and Surveillance Unit, Floor 13, Brunel House, 2 Fitzalan Road, Cardiff CF24 0HA; 5 Macmillan Cancer Support, 89 Albert Embankment, London SE1 7UQ, UK; 6 University College London, Clinical Operational Research Unit, 4 Taviton Street, London WC1H 0BT, UK

**Keywords:** prevalence, survivors, survivorship, UK

## Abstract

**Background:**

Identifying and addressing the requirements of cancer survivors is currently a high priority for the NHS, yet little is known about the population of cancer survivors in the United Kingdom.

**Methods:**

Data from cancer registries in England, Northern Ireland, Scotland and Wales were analysed to provide limited-duration prevalence estimates for 2004. Log-linear regression models were used to extend these to complete prevalence estimates. Trends in prevalence from 2000 to 2004 were used to project complete prevalence estimates forward from 2004 to 2008.

**Results:**

We estimated that in total, there were 2 million cancer survivors in the United Kingdom at the end of 2008, ∼3% of the population overall and 1 in 8 of those aged 65 years and more. Prostate and female breast cancers were the most prevalent. The number of cancer survivors is increasing by ∼3% each year. Estimates are also provided by time since diagnosis.

**Conclusion:**

These estimates are the most up-to-date available, and as such will be useful for statutory and voluntary sector organisations that are responsible for planning and providing treatment and support to cancer survivors in the United Kingdom.

Cancer survivors are defined as those people living with a diagnosis of cancer from some point in their past. Cancer prevalence is expressed as the number or proportion of cancer survivors in a population at a given point in time. Identifying and addressing the requirements of cancer survivors in England is a high priority in the Cancer Reform Strategy (Department of Health, 2007) and, as a result, the National Cancer Survivorship Initiative was set up in 2008. Similar initiatives are being established in Northern Ireland, Scotland and Wales. However, little is known about the size and demography of the population of cancer survivors in the United Kingdom; the most recent estimate, provided by the EUROPREVAL project (Forman *et al*, 2003), was for 1992.

In this paper, we provide up-to-date estimates of cancer prevalence in the United Kingdom at the end of 2008 using cancer registry data. Cancer survivors may have been recently diagnosed and in active treatment, or they may have survived long enough to be considered cured. However, in our analysis, we do not make such distinctions; once an individual is diagnosed with cancer, he (or she) is considered a survivor until death. We adopt this approach because, first, a diagnosis of cancer may affect a person's life in different ways (mental health, fear of recurrence, financial hardship, relationship issues, etc.), and its effects may be felt for many years after diagnosis. Second, this approach is practical as the currently available cancer registration data do not readily allow survivors to be classified as having active disease, in remission or cured of their cancer.

## Materials and methods

The eight cancer registries in England, together with the national registries in Scotland, Wales and Northern Ireland, provided anonymised records of all registered malignant neoplasms (ICD-10 C00-C97) diagnosed in the residents of those countries, excluding non-melanoma skin cancer (ICD-10 C44) as it is not covered systematically by all registries. Each record included demographic, tumour, diagnosis, follow-up and death details. Data were available for the periods 1971–2004 for England, 1971–2005 for Scotland, 1990–2006 for Wales and 1993–2006 for Northern Ireland. All tumours apparently diagnosed in patients over the age of 99 years were excluded (∼0.04% of the total), leaving 7.7 million registration records for analysis.

The UK cancer registries receive death notifications from the Office for National Statistics (ONS) (England and Wales) and the General Register Offices (Scotland and Northern Ireland), which are then matched to the cancer registration records, although a small percentage are never so matched. The patients associated with these ‘lost-to-follow-up’ registrations are at face value, effectively immortal, resulting in apparent cancer survivors of a much higher age than we know to be likely. The proportion of registrations lost to follow-up in European registries is believed to be <1% (Capocaccia *et al*, 1999), but is unknown in the United Kingdom. Therefore, in computing prevalence, cancer survivors were censored at the age of 105 years.

Cancer prevalence can be expressed as the number of prevalent tumours or the number of prevalent patients. As each patient may, in their lifetime, be diagnosed with more than one tumour, patient prevalence will always be lower than tumour prevalence. This analysis focuses on patient prevalence, and only the first diagnosed malignant neoplasm (excluding non-melanoma skin cancer) in each patient was considered.

Although cancer registry data for Scotland, Wales and Northern Ireland were available for the years 2005 and 2006, for England it was available only up to 2004 at the time of analysis. For this reason, we used the most recent index date common to all data, that is, 31 December 2004. The number of cancer patients alive on this date was counted and disaggregated by country of residence, sex, age group on the index date (0–44, 45–64 and 65+ years), number of years since diagnosis and the following broad groups of cancer diagnoses:

1. colon, rectum and anus (ICD-10 C18-C21),
2. lung, bronchus and trachea (ICD-10 C33-C34),
3. female breast (ICD-10 C50),
4. prostate (ICD-10 C61),
5. all other malignant neoplasms excluding non-melanoma skin cancer (ICD-10 C00-C97 excluding C44 and (1) to (4)).

Death-Certificate-Only (DCO) registrations are those for which the only source of patient/tumour information is the death certificate stating the cause of death. These registrations lack much information, particularly the actual date of diagnosis. An unknown proportion of the DCO registrations since the index date will pertain to patients diagnosed before, and alive on, the index date. We have not attempted to estimate this proportion, and therefore they are not included in our prevalence estimates.

### Complete prevalence

*N*-year limited-duration prevalence counts include only those survivors diagnosed in the last *N* years before the index date. Complete prevalence includes all cancer survivors, regardless of when they were diagnosed. It is not possible to directly count complete prevalence on the basis of registry data, given that no UK cancer registry has been collecting data for a sufficiently long period of time. Instead, we estimated complete prevalence by extrapolating from limited-duration prevalence.

With an index date of 31 December 2004, the available cancer registry data provided 34-year prevalence estimates for England and Scotland, 15-year estimates for Wales and 12-year estimates for Northern Ireland. To extend these limited-duration estimates to complete estimates, a negative binomial regression model with a log-link function was constructed for each type of cancer, sex and age group (0–44, 45–64, 65+ years). The prevalence count on the index date was the response variable, and the predictor variables were country of residence and number of years since diagnosis. Given that our primary objective was to obtain a reasonable estimate for the number of people surviving at least 12 years (Northern Ireland) and 15 years (Wales) beyond diagnosis, data pertaining to recent diagnoses (years since diagnosis ⩽5) were not used in the models. Prostate cancer was treated as a special case and modelled in two stages; first, for years of diagnosis between 1992 and 1999 and second, for all years before 1992. We also included an offset term in all models, defined as the log of the number of people in a given country who could contribute to the prevalence count, taking into account the age group being considered and the fact that years since diagnosis cannot exceed age on the index date. The models were run using the PROC GENMOD procedure in SAS (SAS Institute Inc, Cary, NC, USA).

The validity of the regression models was tested by initially excluding data for Scotland covering years of diagnosis between 1971 and 1992, and by comparing the modelled estimates with the actual data for those years. Furthermore, we compared the published estimated ratios of 15-year prevalence to complete prevalence (the completeness index) in England and Scotland (Forman *et al*, 2003) with those of our own (Table 1).

### Trends

Through an analysis of recent trends in observed cancer prevalence, we projected estimates of complete cancer prevalence in the United Kingdom from 31 December 2004 forward to 2008. We used the combined data from England and Scotland covering diagnoses between 1971 and 2004 to estimate trends in limited-duration prevalence during the years 2000–2004, for each site and sex. Log-linear functions, considered appropriate for short-term projections, were fitted to the trend data and provided estimates of the annual growth in cancer survivor numbers that we expected from 2004 to 2008. The following assumptions were made:

1. the yearly rates of change of cancer prevalence in England and Scotland combined can be applied to each country in the United Kingdom;
2. cancer prevalence in each age group (0–44, 45–64 and 65+ years) is changing at the same rate as overall prevalence;
3. complete prevalence is changing at the same rate as 30-year prevalence.

Estimated prevalence counts were converted to proportions of the population by using the mid-year ONS population estimates for 2007; these were the most recently available estimates and likely to be only slightly lower than the actual population at the end of 2008 (ONS, 2008a).

## Results

Tables 2 and 3 and Figure 2 present complete prevalence – the sum of observed prevalence from the years of diagnosis that were available in our data and modelled prevalence from those that were not. We have indicated by italicised text in Tables 2, 3, 4 those estimates for which more than 20% of the total is derived from modelling. We estimated that by the end of 2008, there were just over 2 million cancer survivors in the United Kingdom (59% women and 41% men), equating to ∼2.7% of the male and 3.8% of the female population. Table 2 shows the variation in prevalence by country and cancer sites. Wales had the most number of cancer survivors per capita (3.1% men and 4.2% women), and Northern Ireland had the fewest (2.4% men and 3.4% women).

**Figure 1:**
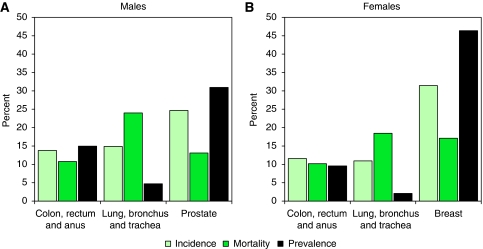
Proportion of total incidence^1^, mortality^2^ and prevalence^3^ that is accounted for by selected cancers. (**A**) Males; (**B**) females. ^1^Incidence in England, 2006; data from National Cancer Information Service (NCIS); ^2^Mortality in England, 2005; data from NCIS; ^3^Prevalence in the United Kingdom, 2008.

Prostate and female breast cancers were the most prevalent, and accounted for 31 and 46% of male and female cancer prevalence, respectively. Of the cancers studied in this paper, lung cancer was the least prevalent. Figure 1 shows, for each sex, the proportions of total incident cases, cancer deaths and cancer survivors that are accounted for by colorectal, lung, prostate and female breast cancers. For both men and women, colorectal cancer accounted for approximately 10–15% of all the three measures. In contrast, for men, lung cancer accounted for 15% of all newly diagnosed cancers, 25% of cancer deaths and for only 5% of cancer prevalence. A similar pattern was seen for female lung cancer, which accounted only for 2% of cancer prevalence in women. Prostate and female breast cancers provided further contrasts, the latter accounting for 31% of newly diagnosed cancers, 17% of cancer deaths and for 46% of cancer prevalence among women.

**Figure 2:**
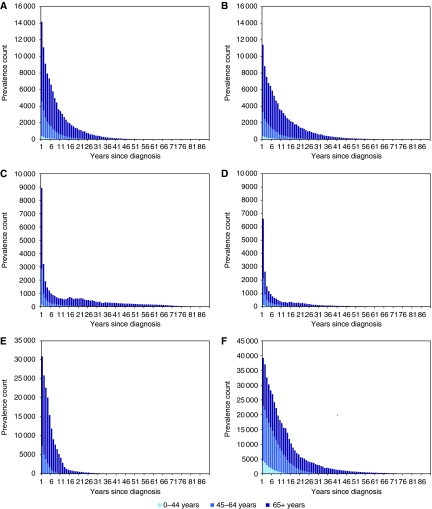
Prevalence of cancer in the United Kingdom on 31 December 2004, by number of years since diagnosis and age. (**A**) Males: colon, rectum and anus; (**B**) females: colon, rectum and anus; (**C**) males: lung, bronchus and trachea; (**D**) females: lung, bronchus and trachea; (**E**) prostate; (**F**) females: breast. Prevalence of cancer in the United Kingdom on 31 December 2004: the sum of observed prevalence was available from national cancer registry data, whereas modelled prevalence was not.

Table 3 presents cancer prevalence in the United Kingdom, by the number of years that had passed since diagnosis. This varied across the sexes and cancer sites, as illustrated in Figure 2. Overall, female cancer survivors tended to be further from their diagnosis than males, 67% of them being diagnosed more than 5 years earlier, compared with 55% of males.

Table 4 shows the variation of cancer prevalence with age. Less than 1% of the UK population aged <45 years at the end of 2008 were cancer survivors, compared with 13% of those aged 65 years and more. There were twice as many female survivors aged between 45 and 64 years as there were males, largely because of the dominance of female breast cancer that accounted for 54% of female survivors in this age range. The most prevalent types of cancer in those aged 65 years and more were prostate and female breast cancers; 5% of males and 6% of females in this age group were survivors of these cancers.

**Figure 3:**
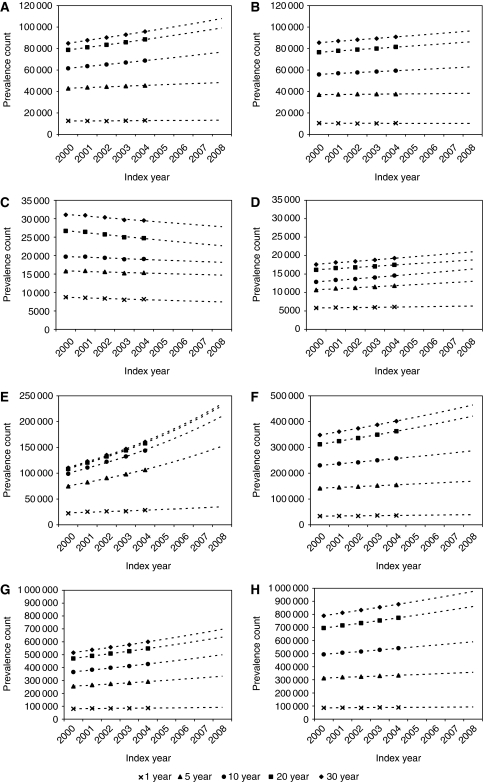
Trends and projections of limited-duration cancer prevalence in the United Kingdom, 2000–2008. (**A**) Males: colon, rectum and anus; (**B**) females: colon, rectum and anus; (**C**) males: lung, bronchus and trachea; (**D**) females: lung, bronchus and trachea; (**E**) prostate; (**F**) females: breast; (**G**) All malignant neoplasms^1^; males. (**H**) All malignant neoplasms^1^; females; ^1^Excluding non-melanoma skin cancer (ICD-10 C44).

Figure 3 shows the trends in 1-, 5-, 10-, 20- and 30-year limited-duration prevalence during the period 2000–2004, projected to 2008. Only male lung cancer did not show an increasing trend, the total number of survivors declining by 1.4% per year. By far, the most rapidly increasing prevalence is that of prostate cancer, the total number of survivors increasing by 9.8% per year. Overall, the number of cancer survivors increased by 3.8% per year for men and 2.7% for women.

## Discussion

For producing our prevalence estimates, we have, where available, used incidence and follow-up data collected by cancer registries in the United Kingdom. We have not adjusted our estimates for DCO registrations that occurred after the index date. DCOs account for <5% of all registrations in the UK and most often relate to patients who have died soon after diagnosis. We have therefore assumed that their effect on cancer prevalence is negligible. We have not attempted to estimate the proportion lost to follow-up nor emigrations. Nor have we been able to include UK immigrants with a diagnosis of cancer pre-dating their move. To a certain extent, the effects of including emigrants and excluding immigrants will cancel each other out.

For estimating the number of survivors from a period before cancer registration in their country, we have developed log-linear regression models for the prevalence count as a function of time since diagnosis. Treating prevalence in this manner as an isolated statistic does not explicitly model the joint effect of incidence and survival, and is based on the observation that the relationship between the number of years since diagnosis and the number of prevalent cases is approximately log linear (Phillips *et al*, 2002). However, as this relationship is not log linear for prostate cancer, the regression model was applied in two stages. This accounted for the introduction of PSA testing in the early 1990s, which effectively changed the definition of the disease with many more localised tumours diagnosed (Evans and Moller, 2003). The number of prostate cancer survivors is increasing at the fastest rate of cancers studied here, by almost 10% each year. As the changes in incidence and survival caused by the introduction of PSA testing are relatively recent, we expect their numbers to increase at a similar rate for some years to come, until a situation is reached in which very few were diagnosed in the era before PSA testing.

Table 1 shows that our estimated 15-year completeness indices for 2004 were consistently lower than those previously published for 1992 (Forman *et al*, 2003). The differences in the completeness index are consistent with the improvements in survival observed between 1992 and 2004 for many cancers (Cancer Research UK, 2007; Rachet *et al*, 2008). Increases in survival rates result in a larger proportion of long-term cancer survivors and therefore, in a smaller completeness index. Although most differences in these indices were small, for male lung cancer, the difference was large (0.58 compared with the published estimate of 0.73). Despite the two estimates relating to different index dates and the changes in lung cancer incidence between the two dates, we would not expect such a large discrepancy. For lung cancer, as with prostate cancer, the number of survivors is not a log-linear function of years since diagnosis. Owing to its poor prognosis, lung cancer prevalence is dominated by short-term survivors (Figure 2c); hence, we believe that our models have over-estimated the number of long-term male survivors. If we were to use a ratio of 0.73, then we would produce an estimate of 6000 (rather than 14 000) male lung cancer survivors who, at the end of 2008, have survived more than 20 years since their diagnosis. This estimate seems more plausible, especially when compared with the report stating that, in the Northern European countries in 1992, only 31% of male lung cancer prevalence was accounted for by survivors who were more than 10 years from diagnosis Moller *et al* (2003).

We have adopted a pragmatic approach to the modelling used in this study. Owing to the long time series of registry data in the United Kingdom, the majority of the estimates presented contain only a small contribution of modelled data. This contribution is most significant in the estimates for Northern Ireland and, to a lesser extent, for Wales. With 34-years of data available for England and Scotland, the modelled proportions of the complete prevalence estimates were typically ∼5% for men and 8% for women. Therefore, we believe these estimates to be robust and fit for the purpose.

### Substantial results of the analysis

There are ∼2 million cancer survivors in the United Kingdom today. In those aged 65 years and more, ∼13% of the population are cancer survivors. Approximately one in three of the UK population will be diagnosed with cancer during their lifetime and one in four will die from it (Cancer Research UK, 2008); we can also state that one in eight of those aged 65 years and more are living with or beyond cancer.

Our overall estimate of 2 million is far higher than that of 1.2 million at the end of 1992 (Forman *et al*, 2003). However, we have found that in recent years the absolute number of cancer survivors in the United Kingdom has increased by ∼3% per annum, and that if a similar rate of increase is assumed to apply over the entire period between 1992 and 2008, then the two figures are consistent. Not only is cancer prevalence increasing overall but the relative prevalence of different types of cancer is also changing. For example, prostate and female breast cancers have shown some of the largest increases in incidence and improvements in survival of all cancers in the United Kingdom since 1992 (Cancer Research UK, 2007), resulting in the proportion of total sex-specific prevalence accounted for by each increasing from 14 and 37% in 1992 (Forman *et al*, 2003) to 31 and 46% in 2008, respectively.

The number of cancer survivors varies within the United Kingdom, with Northern Ireland having the lowest prevalence proportion and Wales the highest. We have not presented age-specific proportions for each country, but the observed differences are, at least in part, attributable to the different age structures in each country; Northern Ireland has the youngest population (63% aged under 45 years; UK average of 59%) and Wales has the oldest population (18% aged 65 years and more; UK average of 16%). Different patterns of adopting PSA testing in the early 1990s resulted in higher detection rates of prostate cancer in Britain, compared with those in Northern Ireland. Consequently, recorded prostate cancer incidence between 1993 and 2003 remained lower in Northern Ireland than in the rest of the United Kingdom (Fitzpatrick *et al*, 2006).

In areas where cancer registration is less comprehensive than in the United Kingdom, models of incidence and survival rates have been developed to estimate cancer prevalence (Capocaccia and De Angelis, 1997). Owing to the large amount of cancer registry data available, little modelling was required, and for simplicity our approach treated prevalence as an isolated statistic. Nevertheless, it is important to appreciate that it is not an isolated measure, and that historical incidence and survival figures combine to produce the prevalence figures existing today. Figure 1 provides an illustration of this interaction: a cancer such as lung cancer, with a poor prognosis accounts for a very small proportion of prevalent cases, despite being one of the most commonly diagnosed cancers. Conversely, prostate and female breast cancers, with relatively good prognoses, account for larger proportions of cancer survivors than they do for new incident cases. Changes in incidence and survival (brought about by changes in lifestyle, population structure and health-service policy) will therefore have significant consequences for the prevalence of cancer. For example, since the 1970s, the number of smokers in the UK male population decreased from ∼50% to ∼20% (ONS, 2008b), resulting in a decrease in male lung cancer incidence and, in turn, a decrease in prevalence.

The previous most recent UK estimates of cancer prevalence were related to 1992 (Forman *et al*, 2003), since then, as we have shown, cancer prevalence has changed markedly. Therefore, our estimates are highly relevant for both statutory and voluntary sector organisations that are responsible for planning and providing treatment and support to cancer survivors in the United Kingdom. In the coming years, cancer prevalence will continue to increase as a result of the growing and ageing population of the United Kingdom, increased detection of cancer and improving survival rates. We estimated that, overall, the annual rate of increase in the number of cancer survivors is currently ∼3%, and we anticipate that this rate of increase will continue in the near future. Issues surrounding care and support for cancer survivors should, therefore, remain high on the public health agenda, with analysis and projections of cancer incidence, prevalence and mortality becoming increasingly central to resource-planning decisions. Cancer detection and treatment resources tend to focus on the most commonly diagnosed types of cancer, or major killers, but for cancer survivors, the most significant cancers are those with both a high incidence rate and a relatively good prognosis (such as prostate cancer and female breast cancer).

Knowledge of the natural progression of a particular type of cancer, together with the analysis of prevalence by time since diagnosis, gives some indication of different phases of survivorship, but does not fully show the extent to which survivors require, or are receiving, care and support. Many survivors will be newly diagnosed and in active treatment, others may be in a state of remission or recurrence with or without late effects of treatment, others may be receiving palliative care, whereas some may consider themselves to be completely free of cancer. Awareness of these differences is important when assessing the health-care burden of cancer, the financial costs of which are the highest during initial treatment and end of life care (Brown *et al*, 1999). We intend to extend the work presented in this paper in order to provide additional details about the different phases of survivorship and to analyse the factors that influence cancer prevalence over time.

## Figures and Tables

**Table 1:** Comparison of 15-year completeness indices, by cancer site and sex

	**Maddams *et al*^a^**	**EUROPREVAL^b^**
*Males*		
Colon, rectum and anus	0.81	0.87^c^
Lung, bronchus and trachea	0.58	0.73^d^
Prostate	0.95	0.97
All malignant neoplasms^e^	0.78	0.82
		
*Females*		
Breast	0.74	0.80
Colon, rectum and anus	0.74	0.80
Lung, bronchus and trachea	0.77	0.79
All malignant neoplasms^e^	0.70	0.72

ICD=International Classification of Diseases.

The 15-year completeness index is defined as 15-year prevalence divided by complete prevalence.

a 15-year prevalence divided by estimated complete prevalence in the United Kingdom. Index date: 31 December 2004.

b Average 15-year completeness index for prevalence as estimated using data from South Thames, West Midlands, Yorkshire and Scotland cancer registries. Index date: 31 December 1992. Published by Forman *et al* (2003), *Ann Oncol* 14: 648–654.

c Average of indices for cancers of the colon and rectum.

d Cancer of the lung only.

e Excluding non-melanoma skin cancer (ICD-10 C44).

**Table 2:** Prevalence of cancer on 31 December 2008 in the United Kingdom, by country of residence

	**England**	**Scotland**	**Wales**	**Northern Ireland**	**United Kingdom**
*Males*					
Colon, rectum and anus	100 608 (401)	11 522 (464)	6921 (476)	*3480* *(404)*	122 531 (410)
Lung, bronchus and trachea	32 034 (128)	3 760 (151)	*1889 (130)*	*1058 (123)*	*38 741* *(130)*
Prostate	215 654 (859)	19 163 (771)	13 312 (916)	5307 (616)	253 436 (847)
All other malignant neoplasms^a^	334 147 (1330)	36 853 (1483)	*22 998 (1582)*	*10 482 (1216)*	404 480 (1352)
All malignant neoplasms^a^	682 443 (2717)	71 298 (2868)	*45 120* (*3103)*	*20 327* (*2358)*	819 188 (2738)
					
*Females*					
Breast	460 041 (1771)	46 211 (1738)	*29 838* (*1955)*	*12 908* (*1439)*	548 998 (1768)
Colon, rectum and anus	92 439 (356)	11 419 (430)	*5885* (*386)*	*3542* (*395)*	113 285 (365)
Lung, bronchus and trachea	19 634 (76)	3215 (121)	1239 (81)	*693* (*77)*	24 781 (80)
All other malignant neoplasms^a^	409 284 (1576)	46 607 (1753)	*26 683* (*1749)*	*13 690* (*1526)*	496 264 (1598)
All malignant neoplasms^a^	981 398 (3778)	107 452 (4042)	*63 645* (*4171)*	*30 833* (*3437)*	1 183 328 (3810)

ICD=International Classification of Diseases.

The number of survivors (crude proportion per 100 000) is indicated; the sum of observed prevalence was available from cancer registry data, whereas modelled prevalence was not. Italicised numbers are those that are based on estimates of prevalence in 2004 that were at least 20% modelled.

a Excluding non-melanoma skin cancer (ICD-10 C44).

**Table 3:** Prevalence of cancer on 31 December 2008 in the United Kingdom, by time since diagnosis

	**0–1 years**	**1–5 years**	**5–10 years**	**10–20 years**	**>20 years**	**Total**
*Males*						
Colon, rectum and anus	14 619 (49)	38 075 (127)	31 162 (104)	24 534 (82)	*14 141* (*47)*	122 531 (410)
Lung, bronchus and trachea	8263 (28)	7850 (26)	3810 (13)	4769 (16)	*14 049*^a^ (*47)*^a^	*38 741* (*130)*
Prostate	37 967 (127)	125 470 (419)	61 376 (205)	22 601 (76)	6022 (20)	253 436 (847)
All other malignant neoplasms^b^	40 891 (137)	97 529 (326)	87 008 (291)	98 726 (330)	*80 326* (*269)*	404 480 (1352)
All malignant neoplasms^b^	101 740 (340)	268 924 (899)	183 356 (613)	150 630 (504)	*114 538* (*383)*	819 188 (2738)
						
*Females*						
Breast	42 432 (137)	140 111 (451)	128 672 (414)	145 035 (467)	*92 748* (*299)*	548 998 (1,768)
Colon, rectum and anus	11 309 (36)	30 341 (98)	27 128 (87)	25 532 (82)	*18 975* (*61)*	113 285 (365)
Lung, bronchus and trachea	6905 (22)	7255 (23)	3671 (12)	2682 (9)	*4268* (*14)*	24 781 (80)
All other malignant neoplasms^b^	40 655 (131)	109 179 (352)	96 400 (310)	119 366 (384)	*130 664* (*421)*	496 264 (1598)
All malignant neoplasms^b^	101 301 (326)	286 886 (924)	255 871 (824)	292 615 (942)	*246 655* (*794)*	1 183 328 (3810)

ICD=International Classification of Diseases.

The number of survivors (crude proportion per 100 000) is indicated; the sum of observed prevalence was available from cancer registry data, whereas modelled prevalence was not. Italicised numbers are those that are based on estimates of prevalence in 2004 that were at least 20% modelled.

a The estimate of the number of long-term male lung cancer survivors (>20 years from diagnosis) is likely over-estimated; see the section ‘Discussion’ for details. A more plausible figure is 6000 (20 per 100 000).

b Excluding non-melanoma skin cancer (ICD-10 C44).

**Table 4:** Prevalence of cancer on 31 December 2008 in the United Kingdom, by age

	**0–44 years**	**45–64 years**	**65+ years**	**Total**
*Males*				
Colon, rectum and anus	2091 (11)	25 690 (343)	94 750 (2238)	122 531 (410)
Lung, bronchus and trachea	441 (2)	6643 (89)	*31 657* (*748)*	*38 741* (*130)*
Prostate	181 (1)	34 511 (461)	218 744 (5168)	253 436 (847)
All other malignant neoplasms^a^	68 539 (377)	125 077 (1671)	210 864 (4982)	404 480 (1352)
All malignant neoplasms^a^	71 252 (392)	191 921 (2563)	556 015 (13 136)	819 188 (2738)
				
*Females*				
Breast	25 428 (143)	208 076 (2694)	315 494 (5688)	548 998 (1768)
Colon, rectum and anus	2134 (12)	19 723 (255)	91 428 (1648)	113 285 (365)
Lung, bronchus and trachea	530 (3)	5904 (76)	18 347 (331)	24 781 (80)
All other malignant neoplasms^a^	67 530 (380)	151 756 (1965)	276 978 (4994)	496 264 (1598)
All malignant neoplasms^a^	95 622 (538)	385 459 (4990)	702 247 (12 661)	1 183 328 (3810)

ICD=International Classification of Diseases.

The number of survivors (crude proportion per 100 000) is indicated; the sum of observed prevalence was available from cancer registry data, whereas modelled prevalence was not. Italicised numbers are those that are based on estimates of prevalence in 2004 that were at least 20% modelled.

a Excluding non-melanoma skin cancer (ICD-10 C44).
